# Urban Place and Health Equity: Critical Issues and Practices

**DOI:** 10.3390/ijerph14020117

**Published:** 2017-01-26

**Authors:** Jason Corburn

**Affiliations:** Department of City and Regional Planning & School of Public Health, University of California Berkeley, Berkeley, CA 94720, USA; jcorburn@berkeley.edu

**Keywords:** urban health, cities, toxic stress, environmental justice, place

## Abstract

Urban places and health equity are two of the most challenging concepts for 21st century environmental health. More people live in cities than at any other time in human history and health inequities are increasing. Health inequities are avoidable differences in the social, environmental and political conditions that shape morbidity and mortality, and disproportionately burden the poor, racial, ethnic and religious minorities and migrants. By linking urban place and health inequities, research and action brings into sharp relief the challenges of achieving urban environmental justice. This article briefly reviews the complex definitions of urban places and how they can shape health equity in cities. I suggest that a more relational or integrated approach to defining urban places and acting on health equity can complement other approaches and improve the ability of public health to meet 21st century challenges. I close with suggestions for research and practice that might focus environmental public health on healthy urban place making. The practices include community driven map making, Health in All Policies (HiAP), promoting urban ecosystem services for health, and participatory and integrated approaches to urban slum upgrading. I conclude that if the global community is serious about the sustainable development goals (SDGs), greater attention must be paid to understanding and acting to improve urban places, living conditions and the social and economic conditions that can promote health equity.

## 1. Introduction

Environmental health happens in places. In other words, exposures to positive or toxic environments occur in geographic locations on the earth that are also sites influenced by human activities and decisions. In 2016, a majority of the world’s population is living in urban environments [1]. Yet, we still know very little about how the multiple characteristics of complex urban environments influence human health.

While I aim to define “urban places” below, health equity entails focused efforts to address avoidable social inequalities by equalizing the conditions for health for all groups, especially for those who have experienced socioeconomic disadvantage or historical injustices [2]. Health equity in this context is not equality (sameness) for all, but rather implies societal efforts to ensure that historically marginalized groups have enhanced opportunities to access health promoting resources and that existing access barriers are removed. In short, health equity means addressing environmental justice, or who gets what and how much, and ensuring openness and fairness in the political processes that make these decisions [2]. Health inequities are increasing in cities and neighborhoods around the world and present one of the greatest equity challenges for urban environmental health [1].

Where you live and how that place is governed can determine when and if you get sick, receive medical treatment and die prematurely [3]. City living can be beneficial for human health, since urban areas generally offer greater economic and educational opportunities, medical services, political and gender rights, affordable housing and cultural, political and religious expression [3]. This holds true in both rich and poor cities of the global North and South. Yet, not everyone in cities can take advantage of these socially produced resources and the poor and socially marginalized often experience health inequities, or differences in access to health promoting resources that are unnecessary, avoidable and unfair. Disadvantaged populations often live in segregated, economically marginalized communities that lack resources for good health and are characterized by poor quality housing, environmental pollution and high rates of crime. As the UN-Habitat and World Health Organization (WHO) stated in their 2010 report, Hidden Cities: Unmasking and Overcoming Health Inequities in Urban Settings:

“*Health inequities are the result of the circumstances in which people grow, live, work and age, and the health systems they can access, which in turn are shaped by broader political, social and economic forces. They are not distributed randomly, but rather show a consistent pattern across the population, often by socioeconomic status or geographical location. No city—large or small, rich or poor, east or west, north or south—has been shown to be immune to the problem of health inequity*.”

[4]

While city living is the quintessential 21st century environment, public health continues to largely focus on studying biologic pathogens, individual behaviors and single environmental exposures (i.e., one air pollutant), but not the complex and often overlapping mixture of environmental hazards and life-supporting characteristics of urban places [5]. What are the multiple environmental exposures—both positive and negative for human health—that exist in today’s cities? How might the characteristics of our places—where we live, learn and play—get into our bodies and influence morbidity and mortality? This article explores some of these questions and the importance of an integrated, or relational, approach to urban places and health equity.

We suggest that a relational approach between urban places and health can help guide research and action in specific ways. For example, a relational approach might help highlight the multiple and often cumulative exposures that burden certain urban population groups. A relational approach to urban place and health equity also suggests that hard to gather spatial data is crucial for good science and policy, and thus engaging residents in processes that identify risks and geographically mapping these can be an important aspect of action-oriented research. We also suggest that a relational approach to policy making can emphasize the importance of a health in all policies (HiAP) framework and strategies of adaptive environmental management and integrated slum upgrading. Together, these practices might offer insights for an integrated approach to understanding and acting to improve urban health equity.

## 2. Urban Places: Beyond the Built Environment

Environmental and urban health scholars tend to frame places as either the “natural” or “built environment” [6]. This dichotomy creates the impression that humans are often “outside” of nature and therefore intrusive in the physical world as they construct habitats, seek and cultivate food, generate energy, and utilize a variety of resources. The nature-built environment binary might also suggests that cities—as human constructs—are destroyers of nature, and by implication, detrimental to all living species dependent on nature for life [7].

A 21st century approach to urban places and health equity ought to do away with strong nature versus urban dichotomies. As William Cronon has argued, idealizing nature often means not valuing and attending to the environments in which most of us now live—namely cities and metropolitan regions [8]. This can contribute to policies that ignore the environmental and public health needs of the urban poor [9]. While not aiming to romanticize urban places and their potential health benefits, we suggest instead that researchers need to understand, value and integrate the complex characteristics of urban places into their work and focus as much on how the multiplicity of place-based factors interact to shape population health. This complexity includes the dynamic interactions between the physical characteristics of urban places and how people and political institutions shape the environments in which we live [10]. A central feature of population health, and one that differentiates it from other models of public health, is that context and features of the built and social environments are understood as key drivers of well-being, not merely the background upon which other mechanisms driving morbidity and mortality take place [11]. Thus, urban places, including the combination of physical, social, political and narrative features and how these interact, should be investigated as a determinant of health.

Urban places can be conceptualized as doubly constructed; through material and physical aspects (the buildings, streets, parks, etc., of the “built environment”) and through the assigning of meanings, interpretations, narratives, perceptions, feelings and imaginations to these places. This is a central feature of the dynamic processes that help define a relational view of place and health [10]. A relational view of urban places suggests that the meanings and ways people interact with their environments are contingent and contested, implying that there is no one single set of place characteristics, meanings or relationships that define a “healthy or unhealthy urban place”. This dynamic also demands that urban health research and practice explore the context-specific differences in social processes, such as power, cultural expression, and collective action, that are often revealed through the construction and reconstruction of environmental assets and hazards, material forms and social meanings in cities and urban areas [12].

This relational view of urban places suggested here can extend the more narrowly conceived variable-centered view of places used in some “neighborhood effects on health”, “built environment and health” and “urban design and health” research today. This research tends to turn characteristics of places, whether physical or social, into covariates in regression models, obscuring the subjective meanings people assign to make sense of their physical and social environments [13]. For example, studies of neighborhood effects on obesity have tended to measure such things as the built environment as a constraint or opportunity for physical activity [14]. While this is likely important, too often these studies ignore the complex interactions of social, environmental and cultural dimensions of why some groups may or may not have the choice to be physically active, such as time spent working multiple jobs due to poverty, lack of street lighting or safety, or historical and cultural narratives that might demean walking in favor of other modes of transportation or activity [15]. Studies of food environments and their influence on diet have also tended to be limited, primarily measuring the number, density and proximity of “healthy” and “unhealthy” food destinations [16]. Economic factors, transportation availability and costs, behaviors that might encourage some to eat and shop for food outside their neighborhood, and the cultural relevance of available food, among other related factors, are less frequently measured [17].

Further, social marginalization is not reducible to single variables, since education, employment, environmental quality, racism, and the affordability and accessibility of other life supporting resources, such as housing, food and social supports, are all multifaceted and interconnected. The often “static” variable-centered approach to urban place and health research misses a key insight from the relational view of urban places, namely that there are mutually reinforcing relationships between places and people, that both context and composition (or one’s biology) matter, as do the institutions and processes that shape one’s physical and social context and access to health promoting resources [7]. Urban place characteristics cannot be neatly compartmentalized if we are to accurately characterize how dynamic living environments influence disease and well-being.

Thus, in a relational view, urban place and health ought to be understood as a result of endogenous and exogenous processes operating at a variety of spatial scales, not just the neighborhood scale [5]. Healey [18] describes the relational approach as emphasizing: “…the dynamic diversity of the complex co-location of multiple webs of relations that transect and intersect across an urban area, each with their own driving dynamics, history and geography...This involves moving beyond an analysis of the spatial patterns of activities as organised in two-dimensional space, the space of a traditional map. Instead, it demands attention to the interplay of economic, socio-cultural, environmental and political/administrative dynamics as these evolve across and within an urban area.”

**Figure 1:**
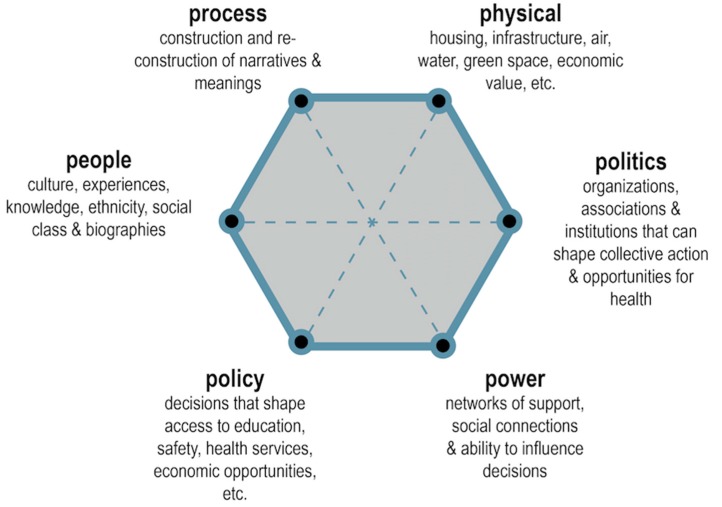
The Relational Framework of Urban Place and Heath Equity.

We have conceptualized the relational view of urban places in Figure 1, which seeks to emphasize not just the nodes (i.e., the “Ps”) but rather how all nodes interact with one another in dynamic, complex ways. We suggest there are at least six intersecting aspects of a relational approach to urban place and health equity research and action. First, an understanding of and engagement with the people living in a community, including valuing their culture, norms, knowledge and history, is essential. Capturing this information in respectful ways will require participatory and democratic methods of inquiry. Second, a relational view of place must understand the physical features in places, here used to capture both human built and natural environments. Ecosystem services in cities, such as tree planting and human-designed green spaces, can offer both ecologic and human health benefits [19]. The physical is also understood as the material and economic values assigned to places, such as tax districts and assessed “property values” which send signals to the market about the economic worth of places and thus influence the local population’s access to capital, services and wealth creation. Third, places are interpreted, narrated, perceived, felt, understood and imagined by people and institutions, and capturing how these narratives are constructed and contested is essential. Fourth, place-making happens within political systems, so capturing how different political organizations—cities, states, national governments, etc.—direct resources and influence decisions in places is another crucial aspect of the relational view. Political associations, organizations, firms, peer-networks and other forms of governance are shaped by and often focused on places, such as neighborhoods, wards, districts, and the like. Fifth, these associations and political systems help shape governance—or the rules, laws and policies—that can distribute or limit resources from reaching people and places, allow or stymie public participation, and ultimately who benefits from public decisions. Sixth, political processes are embedded in, reflect or can confront power relations. Public participation, or the various forms of democratic engagement from voting to organizing to participatory budgeting, is one way the “nodes” of our relational model are linked or integrated. How administrative or electoral districts are delineated, the distribution and location of life-supporting services such as hospitals, and the upkeep or divestment from infrastructure, all reflect the confluence of how people, power, politics, and policies shape processes that distribute physical goods and services in and across urban places (Figure 1).

## 3. Urban Place, Health Equity and Biologic Embodiment

A key challenge in urban place and health research and action is identifying the mechanism(s) through which characteristics of urban places get “into the body”, or are embodied to influence well-being. In environmental health, we might explore the inhalation pathway and the dose-response for, as an example, toxic air pollution. Yet, what if the place-based characteristic is a park or lack of safe streets? Further complicating the dose-response function of urban place characteristics and health is that many communities have multiple, overlapping hazards (and assets) that create cumulative disadvantage/advantage for some over many years or even a life-time.

**Figure 2:**
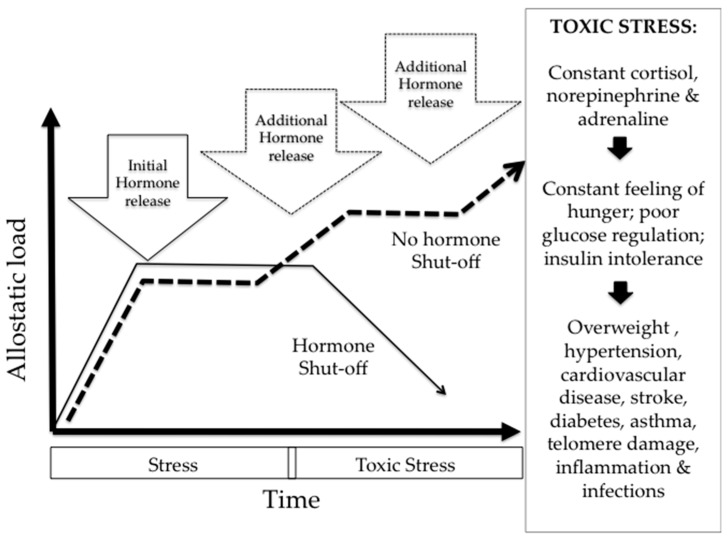
Toxic Stress and related health effects.

The relational perspective described above offers insights for approaching biologic embodiment. For example, as Nancy Krieger has eloquently stated, “a person is not one day African American, another day born low birth weight, another day raised in a home bearing remnants of lead paint, another day subjected to racial discrimination at work (and in a job that does not provide health insurance), and still another day living in a racially segregated neighborhood without a supermarket but with many fast food restaurants. The body does not neatly partition these experiences—all of which may serve to increase risk of uncontrolled hypertension, and some of which may likewise lead to comorbidity, for example, diabetes, thereby further worsening health status” [20] (p. 353). Thus, embodiment research must aim to capture the multiple exposures, and strategies of resilience, that exist in places and population groups [21] (Figure 2).

The concept of toxic stress can help highlight the challenges and approaches for understanding how urban places might influence well-being and health equity. While stress can be life saving for most—think of the fight-or-flight mechanism—constant adversity is toxic, meaning that the prolonged activation of the stress response systems can disrupt the development of the brain architecture and other biologic systems [21].

As we show in Figure 2, under “normal” stressful situations, the human body has a range of physical and chemical responses, but primarily epinephrine (adrenaline) and cortisol are released to bring the endocrine and immune systems back to homeostasis (Figure 2, solid line). The body’s ability to maintain stability under stress has been called allostasis [21]. In toxic stress situations, stressors are constant and the “allostatic load” continues to increase and the chemical release of “fight or flight” hormones does not properly regulate or shut-off (Figure 2, dashed line). Increased allostatic load wears away at the immune system as it overworks to manage the hormonal releases and attempts to return to homeostasis.

Under toxic stress circumstances, the over-secretion of cortisol and adrenaline trigger other biologic responses such as poor glucose regulation and constant feelings of hunger that can contribute to chronic diseases such as overweight and obesity, diabetes, hypertension, cardiovascular disease, stroke, asthma and other immune-related illnesses [22]. Some known toxic stressors include chronic poverty, racial, gender and other forms of discrimination and marginalization, physical or emotional abuse, exposure to violence and housing instability—and these stressors start influencing health in utero and have cumulative impacts over a lifetime [23]. Toxic social stressors over one’s life-course are also suspected of influencing epigenetic processes that regulate whether genes are expressed or suppressed. Allostatic load has been linked with changes in the length of telomeres, which are DNA-protein complexes capping the ends of chromosomes that protect them against damage. Telomere shortening is considered a marker of cellular aging [22]. Capturing the specific features of urban places that might contribute to or mitigate toxic stress is a crucial area of urban environmental public health research and social justice practice [24].

## 4. From Analysis to Practice for Urban Place and Health Equity

### 4.1. Community-Led Mapping

What are some practices that might help capture the complexities of urban places and promote health equity? One example is when communities of the urban poor organize to map the physical and social conditions in which they live and use visualization to advocate for improved well-being [25]. There is a long tradition in urban public health of mapping social conditions, environmental exposures, morbidity and mortality, ranging from John Snow’s historic mapping of cholera cases in 19th century England to 21st century uses of Geographic Information Science (GIS) to analyze environmental exposures across time and space. Community-based organizations (CBOs) are increasingly partnering with universities and others to combine their local knowledge of place with computer aided data collection and mapping tools. For example, in Nairobi, Kenya, slum dwellers have mapped their access to toilets and other life-supporting infrastructure and used these data to demand the human right to safe sanitation and health [26]. In Rio de Janeiro, Brazil, the Mapeamento Digital Liderado por Adolescentes e Jovens (Youth-Led Digital Mapping) was a joint initiative implemented between 2011 and 2015 by UNICEF and a Brazilian non- governmental agency (NGO), the Centro de Promoção da Saúde (Center for Health Promotion, CEDAPS). The project directly involved 550 people in 19 poor communities, where youth mapped socio-environmental health risks as a tool for advocacy and community mobilization [27]. In both the Nairobi and Rio projects, mapping process helped make visible the often ignored or “invisible“ hazards in urban poor communities and these data were used as evidence for health promoting interventions and policies. Community-driven mapping can promote the relational view of urban places by ensuring that often marginalized populations, particularly the urban poor, are leaders in action-research process.

### 4.2. Health Equity in All Urban Polices

Health in all Policies (HiAP) is another important approach to research and decision-making that can highlight the relational characteristics of urban places and their influence on health. HiAP recognizes that most public policies have the potential to influence health, either positively or negatively, but that policy makers outside of the health sector may not be routinely considering the health consequences of their choices and thereby missing opportunities to advance health equity [28]. HiAP is helping governments make the connections between community development and health equity and reveal that non-medical or health care policies can and do shape population well-being [29]. Cities are beginning to adopt HiAP strategies in attempts to understand and shape decisions that influence the multiple dimensions of place-based determinants of health [30]. For example, the City of Richmond, California, became the first in the United States to pass a HiAP Ordinance into law in 2014. Richmond’s HiAP strategy includes reducing inequities across neighborhoods through such urban policies as improving access to and qualities of parks, reducing economic inequities through household energy subsidies, and promoting land use regulations that target food security and reduce climate change vulnerabilities [31]. In northern Spain, a health equity focused impact assessment of a major port redevelopment project incorporated health and equity into the political discourse and, through community-based participatory action, responded to new urban planning challenges by promoting greater health equity [32]. HiAP has the potential to support urban planning and development decisions that promote health equity by shaping place-based resources and redressing historic spatial inequities in cities.

### 4.3. Urban Ecosystem Services and Human Health

As the Sustainable Development Goals (SDGs) make abundantly clear, cities and urban governance are crucial for planetary sustainability [33]. An ecosystem services approach to urban planning can help link health and environmental management decisions. For example, plans to support urban forests and tree planting might help lower urban temperatures, reduce unhealthy heat events and minimize harmful exposures to some air pollutants [34]. Wetland planning in cities might help minimize the adverse effects of flooding and related human health impacts of climate change induced sea-level rise. Environmental management around steep slopes, flood prone riparian zones, and industrial waste sites could all protect the urban poor who disproportionately live on or near these vulnerable land uses [35]. Planning for healthy cities can focus on ensuring that the distribution of health-promoting ecosystem services—including those related to psychological, cognitive, physiologic, and social benefits—reach the poor and most vulnerable communities [36]. Urban social–ecological systems and related human health benefits are complex systems, so this will demand a shift away from purely modeling and predicting future scenarios to more adaptive management that emphasizes flexibility and learning over time [3]. An equitable urban environmental management approach will require participatory experimentation with, not on, vulnerable communities, learning-while-doing, and adjusting interventions and management strategies as new insights emerge from monitoring and tracking [37].

### 4.4. Integrated Participatory Slum Upgrading

A final strategy for healthy and equitable urban place-making is participatory slum upgrading that integrates physical, social and economic improvements for the urban poor [38]. Slums or informal settlements are largely self-built communities and have a range of locally-specific names (such as barrios, bustees, mjondolo, or favelas). Although these areas vary widely in their living conditions and associated environmental health risks, they are rarely recognized officially and typically are denied life-supporting services and infrastructure [39]. Residents of informal settlements typically face multiple risks due to (1) hazardous shelter and local environmental conditions; (2) limited or non-existent access to safe water, sanitation, drainage, public transport, and clean energy; (3) land tenure and housing insecurity; (4) exclusion from affordable, high-quality healthcare, education, refuse collection, and other vital services; (5) spatial segregation; (6) violence and insecurity; and (7) political marginalization [40]. Rather than focus on a single environmental issue or risk, such as clean drinking water or safe housing, integrated or holistic slum upgrading combines multiple objectives into an integrated improvement strategy, co-designed and co-produced by slum residents and professionals [41]. Integrated slum upgrading often addresses the key social determinants of health, including: safety and affordability of housing, services, and infrastructure (including water, sanitation, electricity, roads, footpaths, and refuse collection) [42]; social programs and support for economic development; security of tenure and the right to remain in place; enhanced public spaces and reducing risks of crime; participatory decision-making processes that seek to involve all residents (regardless of tenure status, length of residence, caste or ethnicity etc.), and; integrates the often segregated urban poor communities into the fabric and services of the entire city [41]. For example, an integrated upgrading programme in the Khayelishta township in Cape Town, South Africa, achieved inclusive development through improved public spaces, infrastructure, youth engagement, and social programs, all while reducing violence [43]. In Thailand, the Community Organization Development Initiative (CODI) is a government-community partnership that has achieved integrated urban slum upgrading by supporting and financing community-based groups to design and implement upgrading strategies that have delivered security of tenure, improved infrastructure, social and health improvements [44] Integrated and participatory slum upgrading may be the most important urban health intervention for achieving the Sustainable Development Goals (SDGs) and reducing environmental inequities in cities.

## 5. Conclusions

Healthy and equitable urban places are crucial for achieving health for all populations. Yet, an exclusive focus on either built environments or single risk factors will not address the multiple, often cumulative, social and environmental exposures that exist in cities today and are contributing to inequities disproportionately burdening urban poor communities. A relational perspective of urban places and health equity can help encourage disparate disciplines and institutions to work together, and with the urban poor themselves, to analyze and solve problems. We recognize that this will not be easy and the evidence base for how to do this most effectively is still emerging. Our challenge is great but on an urban planet, environmental public health is urban health, and health equity should be our goal.
